# The Problem of the Low Rates of CRISPR/Cas9-Mediated Knock-ins in Plants: Approaches and Solutions

**DOI:** 10.3390/ijms20133371

**Published:** 2019-07-09

**Authors:** Serge M. Rozov, Natalya V. Permyakova, Elena V. Deineko

**Affiliations:** 1 Institute of Cytology and Genetics, Siberian Branch, Russian Academy of Sciences, Novosibirsk 630090, Russia; 2 Department of Plant Physiology and Biotechnology, Tomsk State University, Tomsk 634050, Russia

**Keywords:** genome editing, gene targeting, knock-in rates, HDR, NHEJ, CRISPR/Cas9

## Abstract

The main number of genome editing events in plant objects obtained during the last decade with the help of specific nucleases zinc finger (ZFN), transcription activator-like effector nucleases (TALEN), and clustered regularly interspaced short palindromic repeats (CRISPR)/Cas are the microindels causing frameshift and subsequent gene knock-out. The knock-ins of genes or their parts, i.e., the insertion of them into a target genome region, are between one and two orders of magnitude less frequent. First and foremost, this is associated with the specific features of the repair systems of higher eukaryotes and the availability of the donor template in accessible proximity during double-strand break (DSB) repair. This review briefs the main repair pathways in plants according to the aspect of their involvement in genome editing. The main methods for increasing the frequency of knock-ins are summarized both along the homologous recombination pathway and non-homologous end joining, which can be used for plant objects.

## 1. Introduction

The development during the last two decades of hybrid nucleases targetable to specific genome sequences, such as FokI, associated with zinc fingers (ZFNs); transcription activator-like effector nucleases (TALENs); and clustered regularly interspaced short palindromic repeats (CRISPR), opened a new era in genetic engineering and biotechnology, the era of genome editing. The CRISPR technology utilizing Cas nucleases (CRISPR/Cas) is the most universal and user-friendly tool for fine targeted genetic manipulations applicable to almost all living species. New notions have emerged, including gene editing, gene targeting and gene therapy. Currently, the *Streptococcus pyogenes* CRISPR/Cas9 system is regarded as the most promising for plant genome editing. In this system, an sgRNA (single guided RNA) recognizes a genome region of 20 bp and the Cas9 nuclease associated with it makes there a double-strand break (DSB) [1,2]. The use of CRISPR/Cas9 toolkit for genome (including plants) editing is comprehensively described in recent reviews [3,4]. Once a DSB is made, cell repair systems start their work; they heal the DNA break according to either HDR (homology-directed repair) or one of the variants of NHEJ (non-homologous end joining) mechanisms. The former provides a precise restoration of the DNA sequence and the latter are error-prone and frequently lead to either replacement of individual nucleotides or small (1–10 bp) indels (insertions or deletions). These indels may cause frameshift and, eventually, lead to knock-outs of the corresponding gene. Similar to the majority of eukaryotes, plants prevalently utilize NHEJ mechanisms for DSB repair [5]. This mechanism function during the overall cell cycle (except for mitosis), whereas HDR acts only in the late S and G2 phases [6].

A concurrent introduction of the plasmid carrying the CRISPR/Cas9 toolkit and a donor template plasmid carrying a particular gene to the cell makes it possible to replace the genome gene with its modified analog or to insert the gene of interest into a target genome region. This precise insertion (knock-in), or gene targeting, demands in most cases, the error-free mechanism provided by HDR. This is especially important for accurate replacement of one gene variant by another. However, this is a rather difficult task since the DSBs in plants are mainly repaired by NHEJ [7]. Indeed, the rate of gene knock-outs resulting from DSB repair according to the NHEJ mechanism is 30–70%, reaching 100% in some cases [8] versus the rate of knock-ins, which typically do not exceed several tenths of a percent, or several percent [4,9]. This explains the fact that the events of plant genome editing described in the literature are mainly represented by gene knock-outs whereas the changes resulting from knock-ins are very few [7]. Nonetheless, many challenges of gene targeting demand knock-ins, for example, the replacement of some alleles with others or the insertion of genes into particular genome regions. That is why many laboratories now develop new methods allowing for an increase in the rate of CRISPR/Cas9-mediated knock-ins. The primary problem to be solved when implementing knock-ins is to decide what is more important—the insertion rate or its accuracy. This determines the repair pathway—HDR or one of the NHEJ, for which the used genetic constructs are to be tailored. Here, we attempt to briefly review the main variants of homologous and non-homologous end joining of DSBs in plants and the relevant methods for enhancing the knock-in efficiency developed so far, involving both the HDR and NHEJ mechanisms.

## 2. Pathways of DNA Double-Strand Break Repair in Plants and Their Use for Producing Knock-ins

DNA DSBs are among the most dangerous genetic damages and their incorrect repair may lead to cell death. Similar to animals, plants utilize several DSB repair pathways, which eventually lead to different results. The eukaryotes have at least two additional DSB repair pathways along with the most prevalent HDR and NHEJ (referred to as a canonical, c-NHEJ) mechanisms, namely, microhomology-mediated end joining (MMEJ), also referred to as alternative NHEJ (alt-NHEJ), and single strand annealing (SSA). Unlike HDR, both additional pathways are as highly error-prone as NHEJ. The DSB repair mechanisms involve a large number of enzymes and other protein factors and they are sufficiently well studied. A detailed consideration of the mechanisms underlying the DSB repair pathways is beyond the goal of this review (see reviews [5,10,11,12,13,14]).

**Figure 1:**
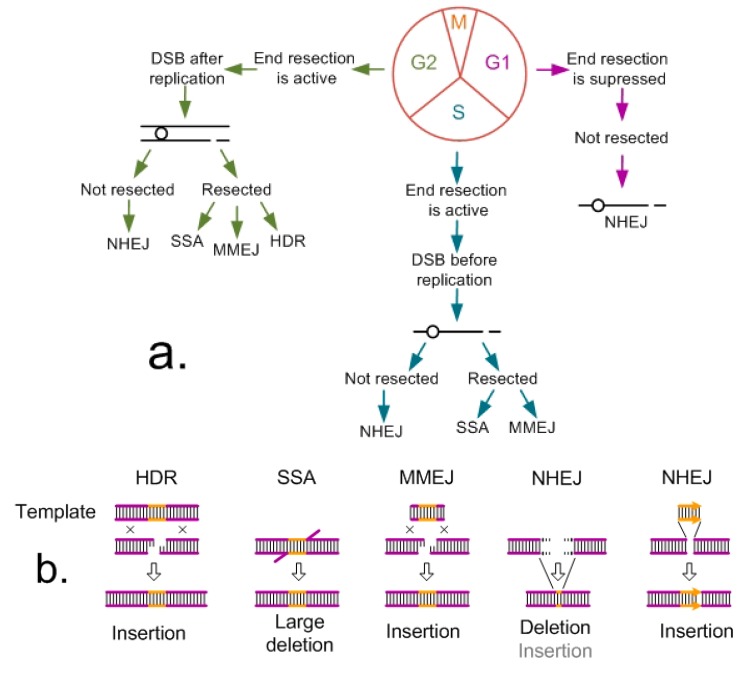
Main mechanisms of double-strand break (DSB) repair in eukaryotes: (**a**) Dependence of the choice of repair pathway on the phase of the cell cycle and (**b**) the results of repair by different mechanisms in the presence and absence of a donor template.

The choice of the pathway for further DSB repair strongly depends on the phase of the cell cycle [14,15]. The meristematic tissues of plants contain actively dividing cells at different stages of the cell cycle, while differentiated tissues consist of the cells that left the cell cycle and stop dividing (G0 phase). At different phases of the cell cycle and in nondividing cells, completely different DSB repair mechanisms may be involved (Figure 1a). NHEJ is active in plant cells during the overall cell cycle except for mitosis (M), when none of the repair mechanisms function in order to prevent telomere fusion. NHEJ is prevalent in the G1 and G2 phases, while MMEJ is most actively utilized in the S phase [16,17,18,19]. Preliminary end resection (formation of 3′ single-stranded ends), activated by cyclin-dependent kinase (CDK), is necessary for the DSB repair according to HDR, SSA, and MMEJ mechanisms. Since the resection of ends in the G1 phase is prohibited, as well as in the G0 of nondividing cells, only NHEJ pathway can be used for DSB repair in G1/G0 [15].

In the dividing cells, HDR is possible only in the late S phase and during G2, when replication is completed and the cell contains sister chromatids. HDR utilizes the homologous sequences of the intact sister chromatid as the template for DSB healing, which provides a complete and precise sequence restoration [18]. As a rule, a donor template flanked with the sequences homologous to the regions at both sides of a DSB is used for precise gene replacement or insertion in the DSB region. The sequence of donor template residing in-between the homologous flanks is precisely inserted into the DSB region by HDR mechanism, leading to a precision knock-in and formation of an insertion of a gene (or its part) or replacement (Figure 1b). As a rule, large insertions require the donor vectors that carry homologous flanking sequences with a length of over 500 bp [14,20].

The resected 3′ single-stranded ends can be joined using small homologous regions of 5–25 nt via the MMEJ mechanism. This pathway is active in the S and G2 phases of the cell cycle. In the absence of a donor vector, this joining leads to small deletions, similar to the consequences of NHEJ. The use of a donor molecule flanked by short homologous sequences makes it possible, in addition to microdeletions, to get knock-ins of the target gene to the corresponding genome region. Since the end joining in this repair pathway is always associated with small deletions, it is purposeful to select the sites for DSB in noncoding or untranslated regions (intergenic spacers or introns) [21,22]. Unlike MMEJ, the end joining according to SSA mechanism requires extended homologous regions and always entails large deletions but not insertions [14,23].

Unlike HDR, the NHEJ-mediated repair does not require a template of the sister chromatid. Although some parts of the NHEJ-repaired sequences do not differ from the intact ones, this pathway frequently generates microdeletions and microinsertions (indels) with a length of one and more nucleotides. That is why NHEJ is frequently used to produce indels in gene-coding regions for their knock-out. However, the NHEJ mechanism, active through the overall cell cycle except for mitosis, in combination with an exogenous donor vector is useful for efficient knock-in generation. Taking into account that NHEJ is highly error-prone, it is better to generate the knock-ins in noncoding or untranslated genome regions [14,24,25], and in the case of gene replacement, this mechanism can create serious problems.

## 3. The Methods Enhancing the Knock-in Efficiency

A tremendous number of genome editing events have been carried out over the last 5 years when the CRISPR/Cas technology is available, which involves animals, plants, and fungi. Unfortunately, the majority of these events are indels, leading to knock-outs of the corresponding genes. Successful knock-ins, i.e., insertions or replacements of a gene variant with another one, are observed at a rate between one and two orders of magnitude lower. This is associated with what most researchers have expected of DSB repair and exogenous DNA insertion according to the HDR pathway, which is unfortunately not prevalent in the higher eukaryotes and is active only for a short time span in the cell cycle. Nonetheless, numerous methods allowing the increase of the knock-in rate and tailored for different DSB repair mechanisms have been designed and proposed during this time. Unfortunately, most of these methods have been elaborated and tested only in animal objects. However, taking into account that the repair mechanisms are in general extremely conserved and similar in both animals and plants, almost all techniques may be used for the plant objects as well. The attempts to increase the knock-in rate in plants are comprehensively considered in several reviews [26,27,28].

### 3.1. Knock-ins Using HDR Pathway

As is mentioned above, the HDR mechanism provides a precise insertion of a donor sequence to a specified region without generating any small indels. That is why this mechanism is most efficient for substituting individual parts of the gene or replacing an allele with another one. The rate of the knock-ins obtained utilizing the HDR pathway can be effectively increased by blocking the NHEJ pathway or by stimulating the HDR mechanism. A *Drosophila melanogaster* mutation leading to a defect DNA ligase IV is shown to increase severalfold the rate of HDR [29]. A knock-out of the *DNA ligase IV* gene severalfold increased the rate of HDR in rice cells [30]. An elevated expression level of some proteins involved in the HDR mechanism can considerably enhance it. In particular, the RAD54 overexpression in *Arabidopsis thaliana* cells tenfold increased the HDR rate there [31]. Numerous experiments with animal cells have shown that Scr7, an inhibitor of DNA ligase IV, increases the HDR rate 2- to 19-fold [32,33,34,35]. Presumably, Scr7 downregulates the expression of the genes coding for other factors involved in the NHEJ pathway (*MRE11*, *DCLRE1C,* and *XRCC4*) [35]. Another approach to enhancing HDR consists in expression inhibition of the genes coding for the key factors of NHEJ pathway. The knock-down of the genes encoding KU70 and KU80 with short interfering RNAs two–threefold decreases the NHEJ rate [32,33]. Thus, an inhibition of the NHEJ pathway can severalfold increase the rate of HDR-mediated knock-ins. However, the danger of this approach consists in that the unrepaired DSBs can accumulate in the cell, eventually leading to its death [36].

Another method to toggle the repair from NHEJ to HDR consists in stimulating the key HDR factors. One of the most important HDR factors is RAD51 [37]. RS-1 stimulates RAD51 synthesis, and two- to four-fold increases the HDR rate, and enhances knock-in efficiency independently of the molecule used as a donor, be it a dsDNA or an ssODN (single-stranded oligodeoxynucleotide). In addition, RS-1 does not interfere with the NHEJ repair pathway [38,39,40]. Overexpression of the *RAD51* gene can also increase the rate of knock-ins up to six-fold [39] although there are some cases when neither RS-1 nor *RAD51* overexpression elevates the HDR rate [41]. Expression activation of the key HDR factors, such as CtIP and CDK1, can two to four-fold elevate the rate of HDR [42]. Another approach consists in construction of a vector carrying the Cas9 gene fused with the CtIP gene or its part [43].

Some small molecules with yet unknown mechanism of their action on DSB repair processes are also useful for increasing the HDR rate. In particular, L755507, a selective antagonist of the human β_3_ adrenergic receptors, can double the HDR rate in mammalian cells in the case of donor dsDNA and increase it up to nine-fold in the case of ssODN [44]. This effect is associated with either the stimulation of key HDR factors or an arrest of the cells in the S phase of the cell cycle [35]. Brefeldin A, a *Penicillium brefeldianum* antiviral factor, doubles the HDR efficiency [44], while small concentrations of resveratrol, present in small amounts in many plants [45], downregulate expression of the key factors in NHEJ pathway (*LIG4*, *MRE11*, and *XRCC4*). It is postulated that the mechanism underlying the resveratrol effect consists in the arrest of cells in the S phase of the cell cycle [35].

The cells in culture also can be synchronized by other chemical agents. For example, nocodazole, which interferes with tubule formation [46], is frequently used to reversibly arrest the cells in the G2/M phases. This approach also increases, two- to six-fold, the rate of the HDR-mediated knock-ins [41,47,48]. Indirubin-3′-monoxime inhibits certain cyclin-dependent kinases and is able to arrest the cell cycle in the G2/M phases, thereby elevating the HDR rate two- to ten-fold. Vinblastine also synchronizes the cells in culture at the G2/M phases by binding to tubulin and blocking the movement of microtubules, thereby increasing the HDR rate six- to ten-fold [49]. The cell synchronization in the S phase can also enhance HDR. In particular, 2′,3′-dideoxycytidine, which slows down replication and considerably elongates the S phase, and hydroxyurea, which inhibits ribonucleotide reductase and arrests the S phase, also increase the HDR rate two- to six-fold [47,50].

Another approach to enhance HDR consists in modification of the Cas9 nuclease itself to make it active in the S/G2 phases of the cell cycle. In particular, the fusion of Cas9 with the N-terminal fragment of the human Geminin (amino acid residues 1–110) resulted in a cell cycle–dependent regulation of Cas9 expression [51]. The fused Cas9–hGem is a substrate for APC/Cdh1, an E3 ubiquitin ligase complex, which efficiently down- and up-regulates the Cas9 expression in the G1 and S/G2 phases, respectively. This approach succeeded in a 1.5-fold increase in the HDR rate. The additional cell synchronization with the help of nocodazole increased the HDR efficiency from 13.8% to 16.2% [51]. It was also shown using fibroblast cell culture that the fused Cas9–hGem doubled the knock-in rate [52]. Thus, Cas9 fusing to expression regulators dependent on the cell cycle phase, has proved promising for increasing the HDR rate.

The rate of knock-ins also considerably depends on the structure of the donor template. DSB repair follows a canonical HDR pathway with dsDNA as a donor and alternative HDR when ssODN is used [53]. When using dsDNA, the knock-in efficiency significantly depends on the lengths of the inserted fragment and the flanking homologous sequences [54]. Short homologous sequences (100–300 bp) considerably decrease the knock-in probability; the highest rate is observable with the flanking sequences of 900–1000 bp long and the efficiency does not grow with the further increase in their length. Extended inserted sequences also decrease the knock-in efficiency [41,54]. Linear rather than circular donor sequences also provide a more efficient HDR recombination [55]. When a plasmid carrying sgRNA binding sites at the outer ends of its homologous arms is used as a donor, it is possible to generate the linear donor directly in the cell with the help of the same Cas9 and thereby to enhance the knock-ins in mammalian cells up to a four-fold higher rate. The main difficulties associated with the use of a double-cut donor consist in that one or both ends can join according to NHEJ, insert together with the homologous flanking sequences, or the remaining part of the plasmid integrate as well [41].

SsODN is a small template (up to 200 nt) for an HDR-mediated knock-in, does not require long homologous flanking sequences, and increases the insertion efficiency. However, this template, being rather short, does not allow knock-ins of extended gene regions and complete genes [56,57]. The best results for the ssODN-assisted knock-ins have been obtained using a template complementary to the strand recognized by sgRNA with a length of 120–130 nt and homologous flanking sequences of 40–90 nt [58,59,60].

A low HDR efficiency can be also associated with the fact that a locus inserted via the HDR mechanism will be again recognized by sgRNA and excised, while the end joining in this case follows a NHEJ mechanism, leading to indels. This problem was solved by using the ssODN with putatively neutral substitutions in the recognition regions for sgRNA and PAM (protospacer-adjacent motif) [61]. This scheme made it possible to avoid repeated cutting and two- to ten-fold elevation in the HDR rate [61]. The knock-in frequency significantly depends on the number and availability of donor templates. A chemical ssODN modification with phosphorothioates improves the stability of the donor sequence and considerably increases the HDR efficiency [62]. Merging Cas9 RNP (ribonucleoprotein) and donor DNA, it is also possible to increase the knock-in frequency. In this case, the donor template automatically appears near the DSB made by Cas9, thereby, the probability of its integration increases. The fusion of the sgRNA and dsDNA donor into one molecule allowed the knock-in efficiency to be increased threefold [63]. A covalent tethering of an ssODN donor template to Cas9 via the PCV (porcine circovirus 2) Rep protein, one of the tyrosine residues of which is able to bind to oligonucleotides, has allowed the HDR-mediated knock-ins to be enhanced 30-fold [64].

### 3.2. NHEJ- and MMEJ-Mediated Knock-ins

Thus, it is evident that a high rate of the HDR-mediated knock-ins is rather difficult to achieve and requires some intricate manipulations that alter the eukaryotic repair systems. Correspondingly, many researchers try to utilize the MMEJ and, especially, NHEJ (which is the main repair pathway in higher eukaryotes) mechanisms to increase the knock-in rate of exogenous DNA.

A new technique for the NHEJ-mediated insertion of exogenous DNA into a target locus, referred to as obligate ligation-gated recombination (ObLiGaRe), was designed utilizing closely located sites for a ZFN in the intron of a target gene [65]. The same recognition sites were inserted into the donor plasmid. The nuclease made DSBs not only in the target site, but also concurrently in the donor plasmid, producing a linear dsDNA. During a NHEJ-mediated end joining, this 15-kbp dsDNA with a relatively high probability inserted in the target site. The efficiency of this ObLiGaRe technique appeared to be higher as compared with an HDR-mediated insertion. However, the sites of end joining in this case frequently carry small indels since the integration follows an NHEJ mechanism [65].

A similar technique for enhancing the NHEJ-mediated knock-ins in a target genome locus, named homology-independent targeted integration (HITI), was successfully used quite recently [25]. The gene coding for the fluorescent protein IRESmCherry was inserted into the coding sequence of the earlier integrated *GFP* gene in several variants. In one case, the donor plasmid carrying the *IRESmCherry* gene did not have any site for sgRNA recognition identical to the site in the *GFP* target; in the second variant, it carried one site in question; and in the third variant, *IRESmCherry* was flanked from both sides with these recognition sites. In the last two cases, Cas9 cuts the donor plasmid to give a linear dsDNA molecule. IRES (internal ribosome entry site) was used to avoid the consequences of possible frameshift, emerging as a result of microindels during an error-prone NHEJ repair. In the experiments with different mammalian cells, the frequency of HITI-mediated knock-ins increased several tenfold. It is difficult to quantitatively assess the degree of this increase since any HDR-mediated insertions were in most cases unobservable in this study [25].

A similar system comprising Cas9 and two different sgRNAs was recently used; in this system, a nuclease with one sgRNA generated a DSB in the target site and with the other sgRNA concurrently cut the donor plasmid to produce linear dsDNA [66]. The ends were joined by the NHEJ mechanism and the knock-in rate was considerably increased although the small indels were frequently observed at the sites of end joining [66]. This integration method can also work using one sgRNA if the sequence(s) identical to the sgRNA binding sites of the target locus are introduced before or even at both sides of the expression cassette in the donor plasmid. This makes it possible to avoid integration of the unnecessary part of the plasmid sequence into the genome. This technique was earlier used to insert the *GFP* gene to a target wheat locus according to NHEJ mechanism using the TALEN toolkit [67].

NHEJ-mediated knock-ins were efficiently generated in mammalian cells using rather extended ssODN sequences as a donor template [68]. In this case, long fragments were integrated at a lower rate (~4%), while the efficiency of relatively short knock-ins reached 17% [69].

Another method for MMEJ-mediated integration of exogenous sequence into the genome, named PITCh (precise integration into target chromosome), utilizes both the TALEN and CRISPR/Cas9 toolkits [22,68]. In the second (CRISPR/Cas9) variant, the system uses three different sgRNAs, one of which generates a DSB in the target sequence, and the two others cut off the linear dsDNA to be inserted from a template vector. The ends of this dsDNA carry the regions of microhomology (5–25 bp) to the sequences flanking the DSB target and the knock-in is formed by MMEJ mechanism [22,68].

Thus, both NHEJ and MMEJ mechanisms are promising for generating knock-ins more efficiently as compared with the HDR-dependent variant; however, error-prone DNA joining at the sites of insertion still remain its main disadvantage and may cause problems in the case of precise gene replacement.

### 3.3. Other Approaches to Increasing Knock-in Efficiency

A high knock-in efficiency (both for gene replacement and gene insertion) requires that the cell simultaneously contains sufficient amounts of Cas9/sgRNA RNP complex and the donor template. When they are concurrently delivered to a plant cell by biolistics within different plasmids or by agrobacterial transformation in one plasmid, the Cas9 endonuclease activity reaches its maximum only on day three after the transformation. However, no effective amounts of the donor template are likely to remain in the cell during this time owing to the endogenous nuclease activities. If the in vitro assembled ready-to-use Cas9/sgRNA RNP complex is delivered to the cell by biolistics simultaneously with the donor template, its activity will be maximum during the first 12–24 h [70]. A repeated transformation with the donor template after 2–3 days is senseless since the probability of hitting the same cells for the second time is extremely low. That is why a biolistic delivery of the preassembled Cas9/sgRNA RNP complex simultaneously with the donor template is regarded most promising for efficient knock-ins in plant cells [71,72]. In addition, a long-term expression of the Cas9/sgRNA RNP complex in the cell may bring about accumulation of undesirable off-targeting events [70]. The Cas9 delivery as a preassembled RNP that has shown increased knock-in rates in lettuce [73], maize embryos [71], and wheat [74].

An approach opposite in its essence has been elaborated for *Arabidopsis thaliana* and demonstrated its efficiency for increasing the knock-in frequency [75]. Initially, the authors tried the transformation using an all-in-one plasmid and demonstrated that this approach was completely inefficient and then tested a sequential transformation. First, they obtained the plants that stably expressed Cas9 under the control of different promoters and then repeated an agrobacterial transformation using the corresponding sgRNA and donor template. The resulting knock-in rate reached 5–9%. Interestingly, this was observed only with the *A. thaliana* line where Cas9 was transcribed under the control of DD45 promoter, specific for the egg cells and early embryonic stage [75].

Another approach increasingly multiplies the amounts of Cas9/sgRNA and donor template in the plant cell, is their integration in modified geminiviral vectors. Geminiviruses are a large family of plant viruses with a single-stranded circular DNA genome (~2.5–3.0 kb) affecting both dicot and monocot plants. When infecting cells, they produce a large number of replicons via a rolling-circle replication. Such modified replicons can be used to rapidly synthesize a large number of repair templates in the plant cell. This approach allows the knock-in frequency to be increased 10- to 100-fold [9,76,77]. It is sufficient to assemble a construct comprising the coding region of replication initiator protein (Rep/RepA), large intergenic region (LIR), and short intergenic region (SIR) and supplement it with the necessary repair template sequences. The assembled replicon can be delivered to the cell by biolistics or agrobacterial transformation. This method has allowed for targeted knock-ins in tobacco and tomatoes [78,79]. The replicons of the bean yellow dwarf virus (BeYDV) are most frequently used for increasing the knock-in rate in dicots and wheat dwarf virus (WDV), in monocots [28].

## 4. Conclusions

Hundreds and even perhaps thousands of events of targeted genome editing have been performed over the last decade. Unfortunately, the major part of these events is gene knock-outs, whereas knock-ins (both gene insertions and gene replacements) are by one to two orders of magnitude less frequent. Development of approaches to solve this problem are in progress; however, there is still no universal and efficient method for increasing the knock-in frequency. Unfortunately, most studies in this area involve mammalian cells and many already available techniques have not been tested in plant objects. Taking into account that knock-ins are implemented by the repair systems, which are most conserved for all eukaryotes, the methods developed for animals are likely to work for plants as well. Here we tried to brief the most recent efficient efforts in this area that are applicable to plant objects. It is clear that this problem is multifactorial and demands a multistep solution. Nonetheless, some general trends have become evident, which are likely to lead to success in future. It is clear now that the NHEJ pathway is more promising as compared with HDR although it does not always provide the necessary precision, required, for example, for gene substitution. However, the effects of microindels are resolvable by, for example, utilization of additional IRES elements. Another trend is associated with the structure of donor templates. It is ever more evident that the knock-in efficiency increases when the donor template is a linear dsDNA but the cut-off must be simultaneous with the DSB generation in the target site. Moreover, the use of ssODNs as a donor template in several cases is more successful. Most likely, the knock-in generation in plant objects will soon become a routine procedure owing to a combination of different approaches, manipulations with the repair machinery, and modifications of the repair template structure.
